# Supplementary material for: NEK6 Regulates Redox Balance and DNA Damage Response in DU-145 Prostate Cancer Cells
Source: Cells. 2023 Jan 7;12(2):256. doi: 10.3390/cells12020256 (PMC9856815; doi:10.3390/cells12020256)

Supplementary figure S1 (Figure S1). Detection of up to four indels in NEK6-KO cell lines by Sanger-type sequencing.

NEK6-KO (83.7)

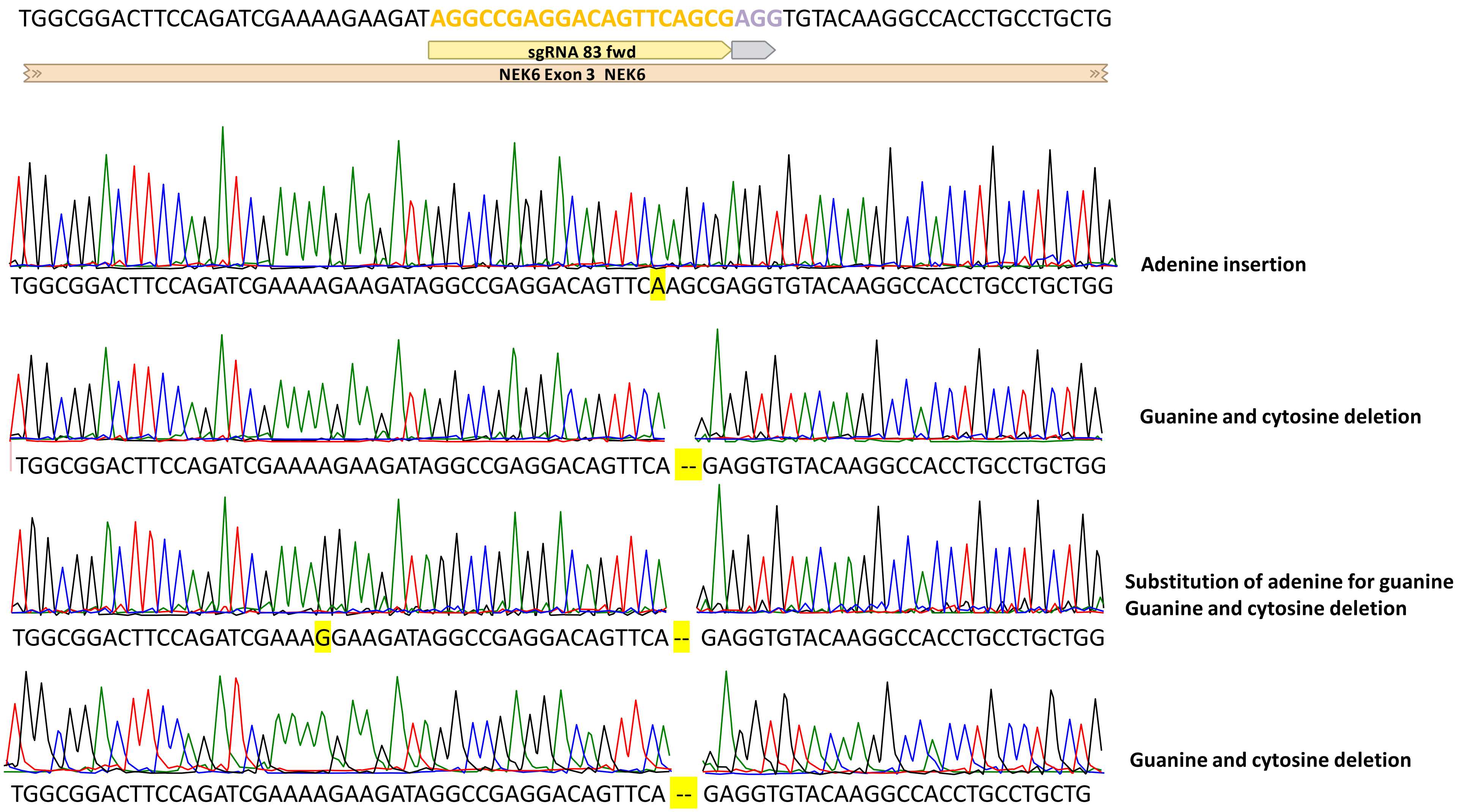

Figure S1 (cont.)

NEK6-KO (83.14)

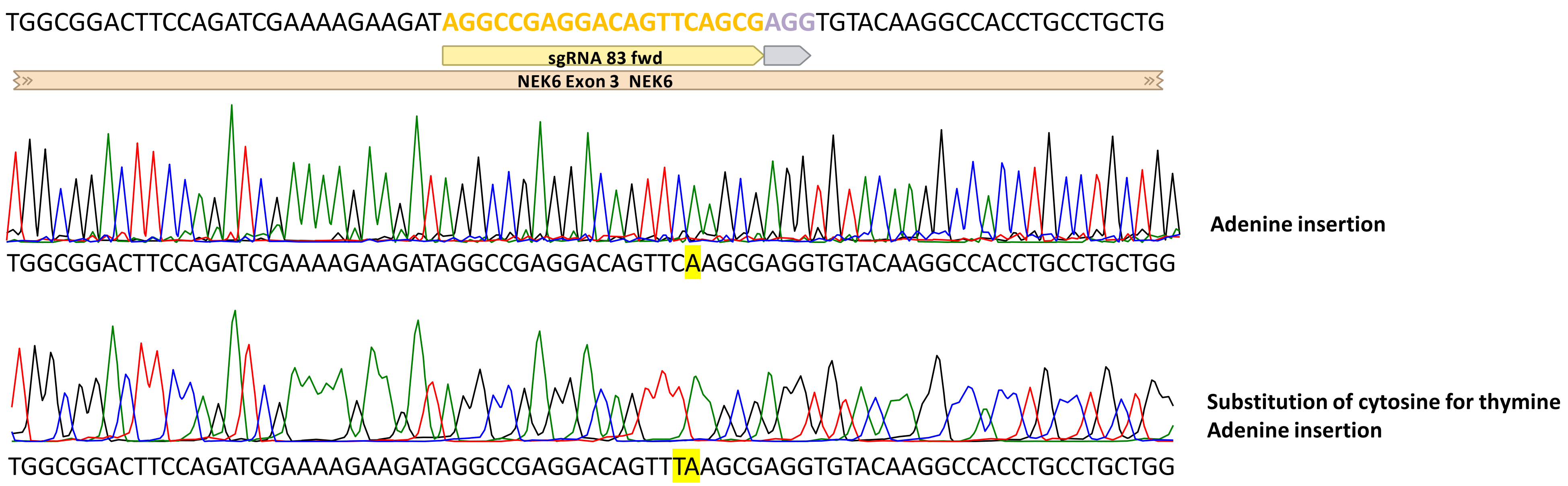

Figure S1 (cont.)

NEK6-KO (56.3)

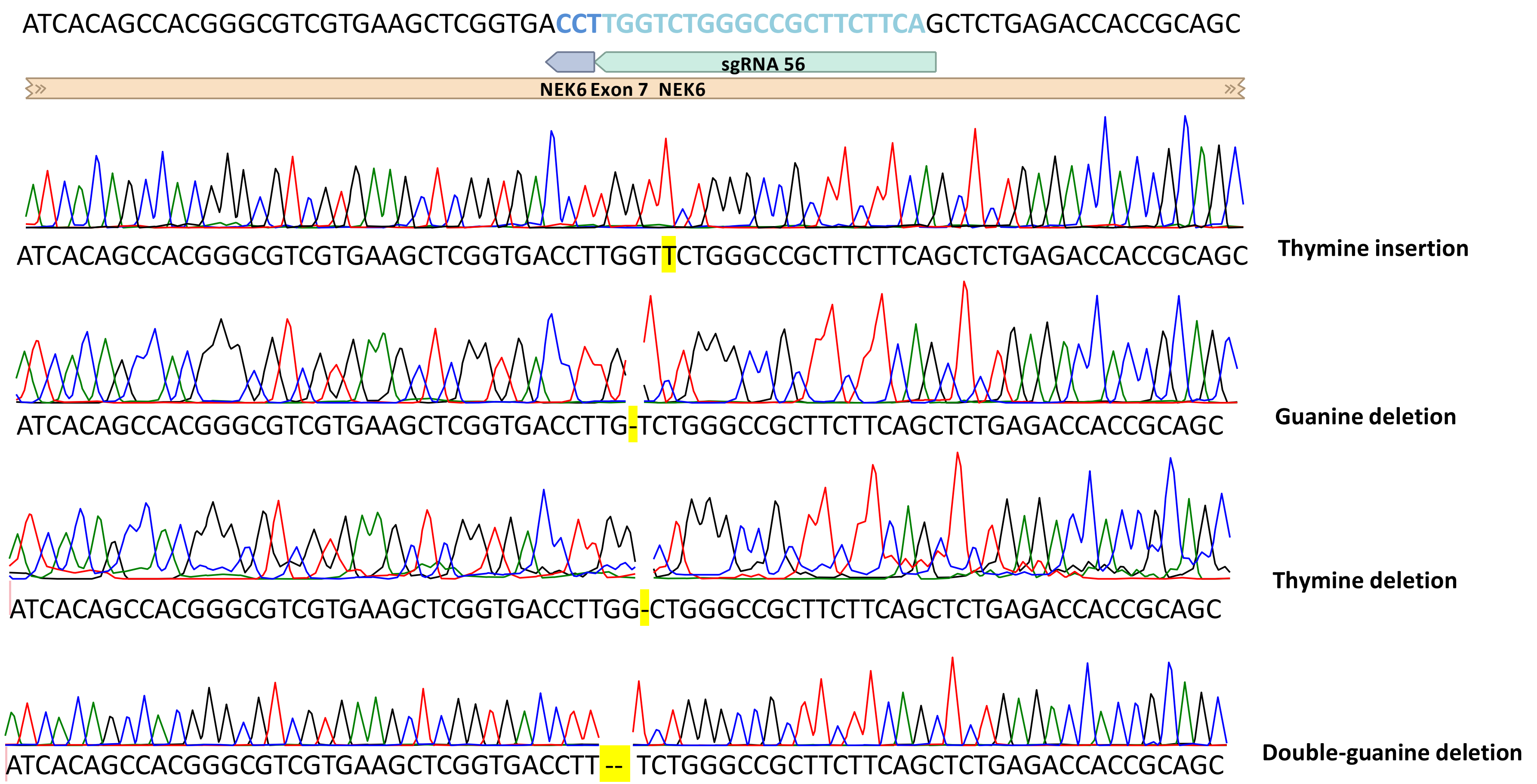

Figure S1 (cont.)

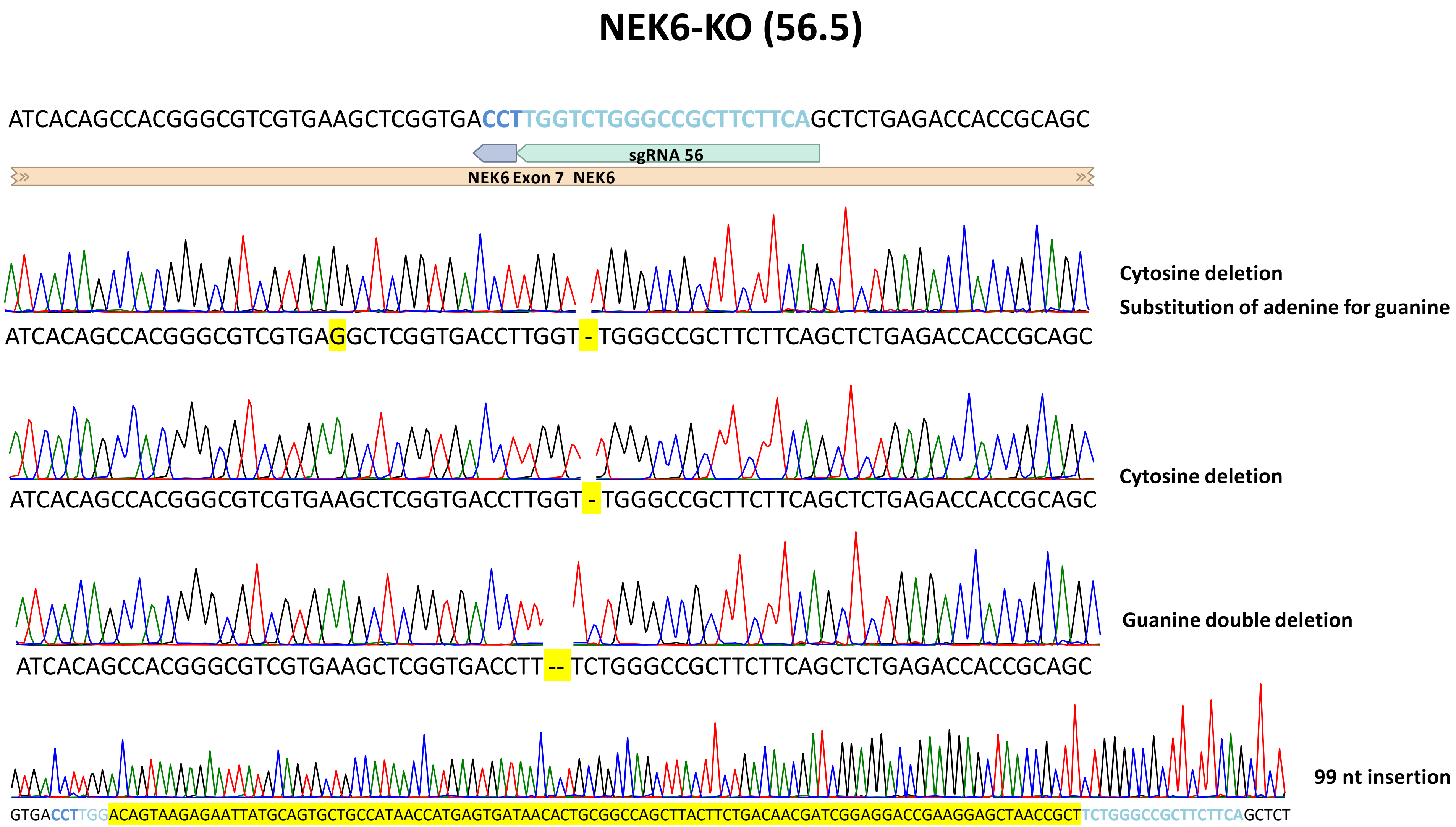

Supplementary figure S2 (Figure S2). Knockout of NEK6 alters NEK2, NEK7, NEK9, and NEK11 expression.

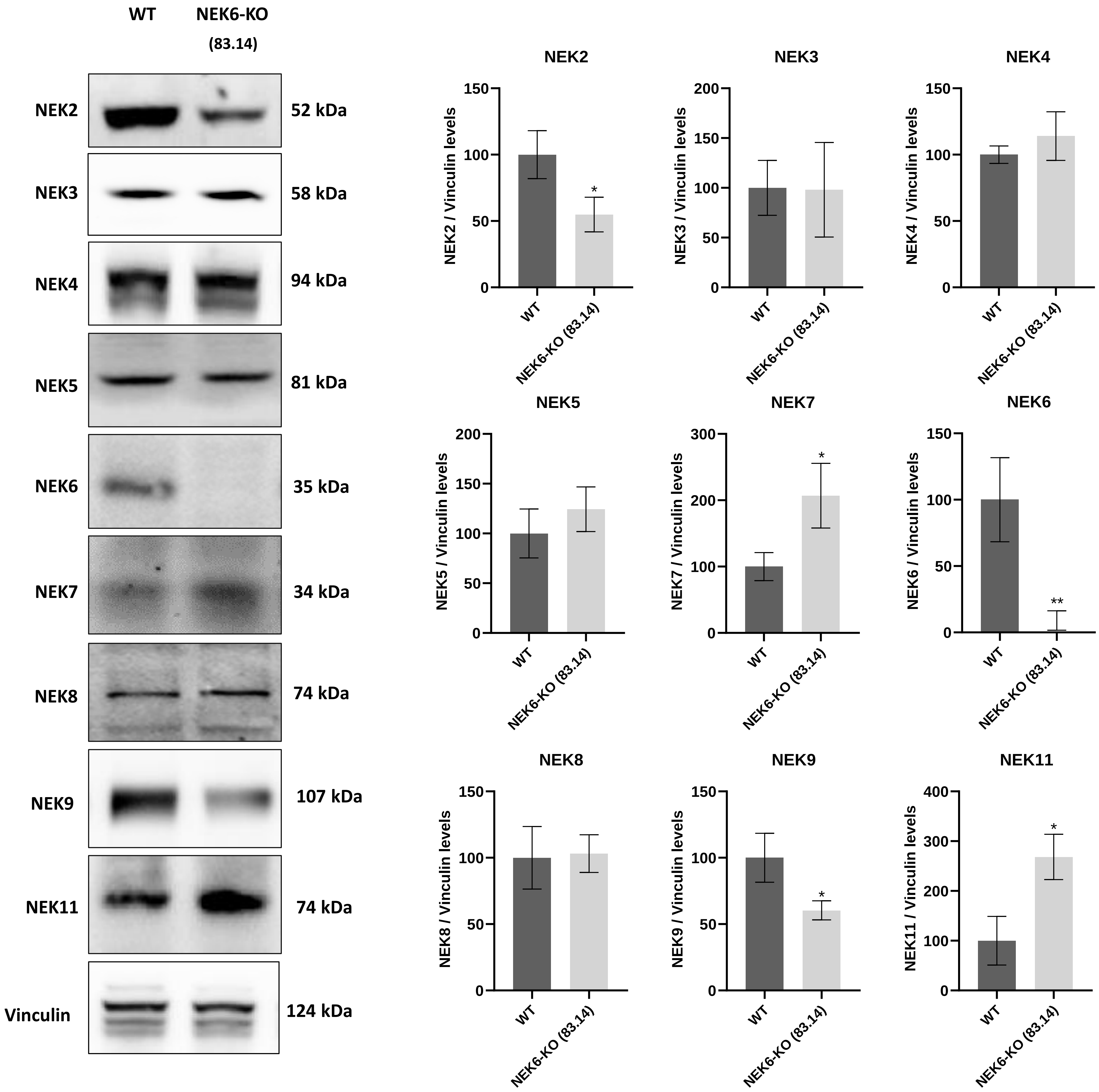

Supplement: Supplementary file 1 [file cells-12-00256-s001.zip › cells-2026683-supplementary.pdf]
